# FDNet: Knowledge and Data Fusion-Driven Deep Neural Network for Coal Burst Prediction

**DOI:** 10.3390/s22083088

**Published:** 2022-04-18

**Authors:** Anye Cao, Yaoqi Liu, Xu Yang, Sen Li, Yapeng Liu

**Affiliations:** 1 School of Mines, China University of Mining and Technology, Xuzhou 221116, China; caoanye@163.com; 2 Jiangsu Engineering Laboratory of Mine Earthquake Monitoring and Prevention, China University of Mining and Technology, Xuzhou 221116, China; 3 Xuzhou Wushuo Information Co., Ltd., Xuzhou 221116, China; 4 School of Computer Science and Technology, China University of Mining and Technology, Xuzhou 221116, China; 08182754@cumt.edu.cn (S.L.); ts21170016a31@cumt.edu.cn (Y.L.)

**Keywords:** coal burst, coal mine safety, fusion-driven, deep neural network

## Abstract

Coal burst prediction is an important research hotspot in coal mine production safety. This paper presents FDNet, which is a knowledge and data fusion-driven deep neural network for coal burst prediction. The main idea of FDNet is to extract explicit features based on the existing mine seismic physical model and utilize deep learning to automatically extract the implicit features of mine microseismic data. The key innovations of FDNet include an expert knowledge indicator selection method based on a subset search strategy, a mine microseismic data extraction method based on a deep convolutional neural network, and a feature deep fusion method of mine microseismic data based on an attention mechanism. We conducted a set of engineering experiments in Gaojiapu Coal Mine to evaluate the performance of FDNet. The results show that compared with the state-of-the-art data-driven machines and knowledge-driven methods, the prediction accuracy of FDNet is improved by 5% and 16%, respectively.

## 1. Introduction

Coal burst is a common geological disaster in coal mining. Most mining countries, such as Canada, the United States, Germany, and Australia, have recorded coal bursts [1,2,3]. The cause of the disaster is that when the stress accumulation of the coal and rock mass exceeds its strength limit, the elastic energy is released instantaneously, resulting in the instantaneous destruction of the coal and rock mass [4,5,6,7]. Accompanied by a large amount of coal and rock mass gushing out, coal bursts cause casualties and equipment damage in coal mines [8]. From 2018 to 2021, coal mines in China suffered multiple coal burst accidents, resulting in around 20 deaths. Therefore, coal burst monitoring and early warning is the current research hotspot.

The prediction and early warning of coal bursts are essential to monitor some precursory signals of coal bursts during the construction stage utilizing the electromagnetic radiation method, micro-gravity method, infrared thermal imaging method, and microseismic monitoring; then, early warnings of the occurrence of coal bursts are possible. Microseismic monitoring is one of the most widely used early warning methods [9,10,11]. Typically, several microseismic events precede the onset of coal bursts, and these events record precursory information about coal fracture and stress transfer. Microseismic monitoring can determine the time, location, and intensity of these microseismic events in real time, thereby predicting the occurrence of coal bursts [12,13].

The research work of coal burst prediction using microseismic data can be divided into two categories: knowledge-driven and data-driven. The knowledge-driven coal burst prediction method uses geophysics to establish a variety of indicators (using frequency, energy, *b* value, A(b) value, etc.) to build a coal burst prediction model [14,15,16,17]. However, such methods cannot fully obtain the sequence information of mine microseismic data, resulting in poor recognition accuracy. Drawing on the successful experience of earthquake prediction [18,19,20,21], in recent years, there has been much research work on data-driven coal burst prediction based on machine learning [22,23,24]. Among them, deep learning has a good application prospect in coal burst prediction because it can automatically extract the implicit features of massive mine microseismic data [25,26,27]. However, this kind of work only starts from the perspective of statistical data, without integrating the existing expert knowledge, and the recognition accuracy needs to be further improved. In addition, the data-driven method also has problems such as poor interpretability.

To address the above issues, this paper proposes a novel knowledge and data fusion-driven deep neural network for coal burst prediction, called FDNet. The main function of FDNet is to use the microseismic data to predict whether a large-energy coal burst will occur in the next 1–3 days. The main idea is to extract explicit features based on the existing mine seismic physical model, use deep learning to automatically extract the implicit features of mine microseismic data, and then build a coal burst prediction method based on the fusion features.

Building the above model mainly faces the following key challenges: (1) how to select specific expert knowledge indicators for different coal mines; (2) how to extract implicit features from massive mine seismic data; (3) how to deeply integrate knowledge-driven and data-driven extracted features. To address the first challenge, we design an expert knowledge indicator selection method based on a subset search strategy to solve the multi-indicator screening problem of mining microseismic data. To address the second challenge, we establish a mine microseismic data extraction method based on a deep convolutional neural network, realizing the implicit feature extraction of massive mine microseismic data. To address the third challenge, we propose a feature deep fusion method of mine microseismic data based on an attention mechanism, which realizes the feature fusion based on knowledge-driven and data-driven.

The main contributions of this paper are summarized as follows.

- To the best of our knowledge, this is the first study of a knowledge and data fusion-driven deep neural network for coal burst prediction. This network utilizes the fusion features of expert knowledge and data-hidden information.
- We establish a novel mine microseismic data extraction method based on a deep convolutional neural network and propose a novel feature deep fusion method of mine microseismic data based on an attention mechanism.
- We have conducted a set of engineering experiments in Gaojiapu Coal Mine to evaluate the performance of FDNet. The results show that compared with the state-of-the-art data-driven and knowledge-driven methods, the prediction accuracy of FDNet is improved by 5% and 16%, respectively.

## 2. Overview

**Figure 1:**
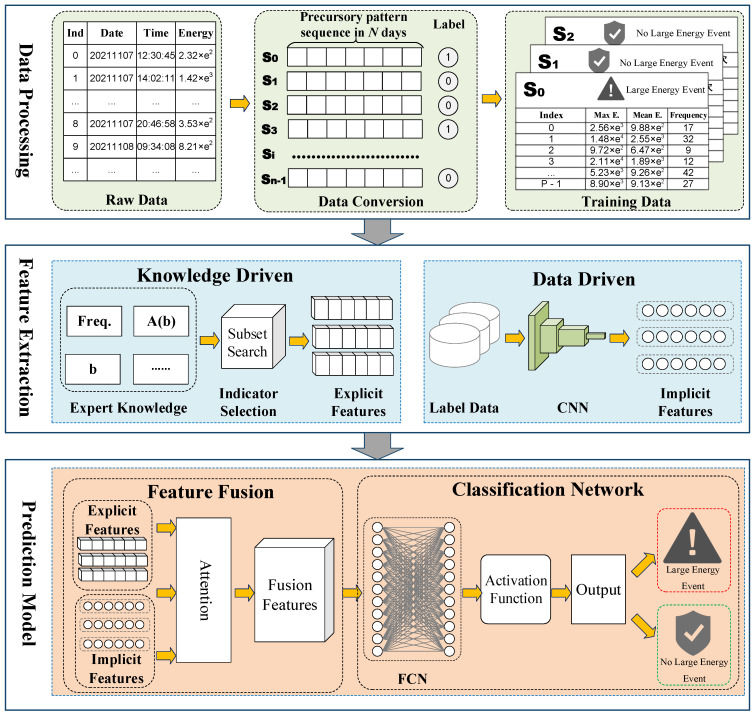
Knowledge and data fusion-driven deep neural network architecture.

As shown in Figure 1, the research in this paper mainly includes three parts: mine microseismic data processing, feature extraction, and prediction module.

Mine microseismic data processing converts the raw data collected by microseismic sensors into precursory pattern series data for model input. The original data saved by the microseismic system include the time of the microseismic event, the energy of the microseismic event, and the coordinates of the source of the microseismic event. Firstly, the raw data are statistically analyzed, and the daily maximum energy value and mean energy value are calculated to generate time series data with daily as the minimum unit. We specify the precursory pattern sequence length to generate multiple precursory pattern sequences and their labels.

Feature extraction includes knowledge-driven explicit feature extraction and data-driven implicit feature extraction. Explicit features refer to the relevant indicators calculated by expert knowledge, including the *b* value, *a* value, A(b) value, seismic absence, microseismic activity scale ΔF, microseismic time information entropy, equivalent energy level parameter EEM, and microseismic activity *S* value. Aiming at different coal mines, we present a subset search method to select specific indicators to obtain the optimal display characteristics. Implicit features refer to the hidden regularity information mined from massive data using deep learning methods. In this paper, convolutional neural networks are used to achieve implicit feature extraction.

Predictive models include feature fusion and classification networks. In the feature fusion part, we propose a deep fusion method of explicit and implicit features based on the attention mechanism. In the classification network part, we implement classification through fully connected network fitting, thus constructing a prediction module of coal burst large-energy events.

## 3. Detailed Design

### 3.1. Data Processing

**Figure 2:**
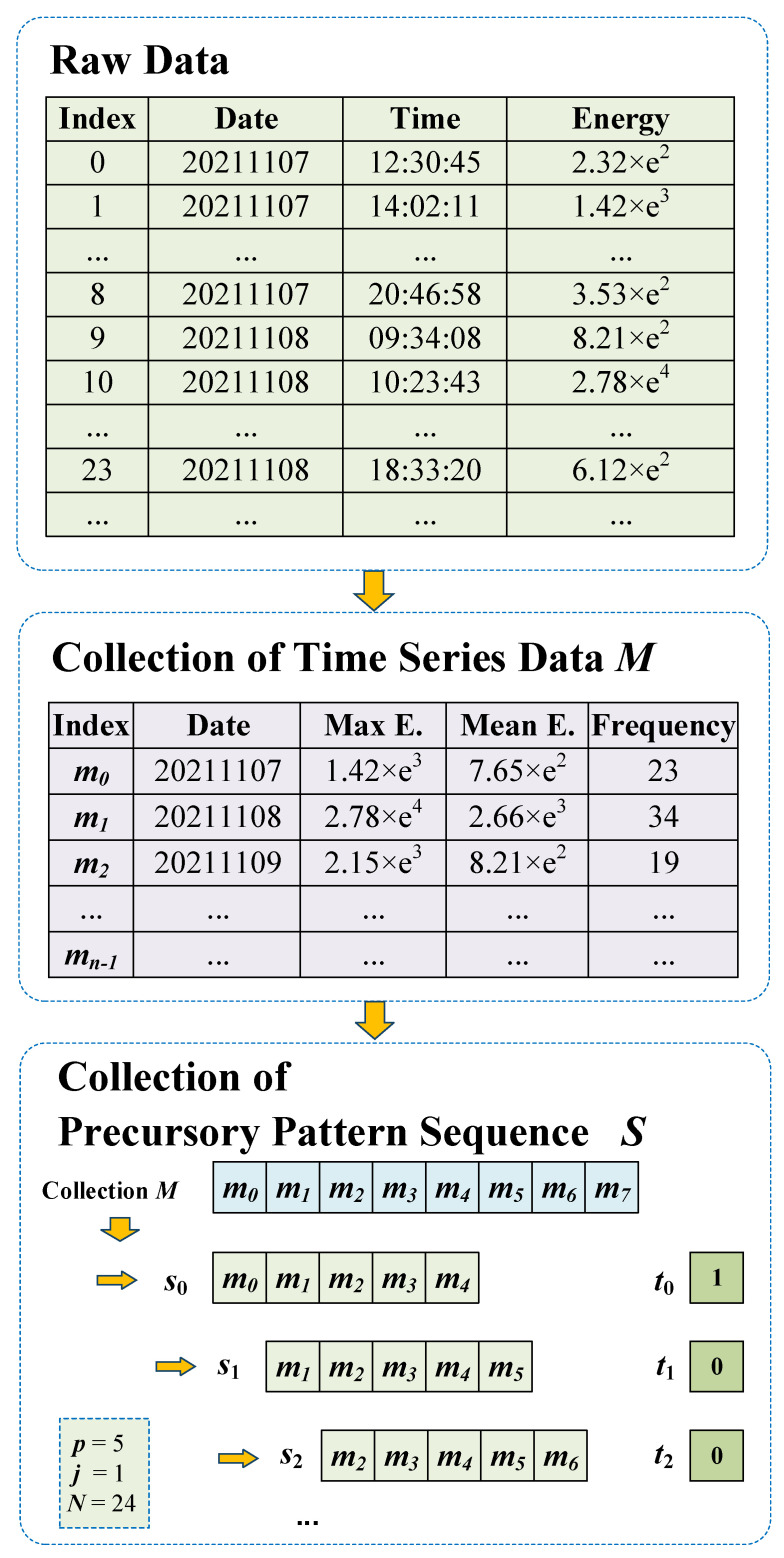
The workflow of data processing.

The original data of the database contain information such as microseismic time, microseismic energy, and source coordinates. As shown in Figure 2, the following processing needs to be done on the raw data to be suitable for the training and prediction of the model.

First, we build a time series data set with fixed time window statistics. Assuming that the data record calculated in the *i*th time window is $m_{i}$, it can be expressed as:

(1) $$m_{i}=\langle id,e_{max},e_{mean},f\rangle$$

where id is the time window number, $e_{max}$ is the maximum energy in the time window, $e_{mean}$ is the mean energy in the time window, and *f* is the frequency of microseismic events within the time window. Therefore, when the data are divided into n time windows, traversing the time windows based on the above method, the time series data set can be obtained as $M=\langle m_{0},m_{1},m_{2},\ldots ,m_{n-1}\rangle$.

Then, a precursory pattern sequence is constructed based on the above processed time series data. Assuming that the ith precursory pattern sequence is $s_{i}$, it can be expressed as:

(2) $$s_{i}=\langle m_{i\times j},m_{i\times j+1},m_{i\times j+2},\ldots ,m_{i\times j+p-1}\rangle$$

where *p* is the length of the precursory pattern sequence, and *j* is the sampling step size. Therefore, under the premise of *n* time windows and n≫p, the precursory pattern sequence set can be generated based on the above method:

(3) $$S=\langle s_{0},s_{1},s_{2},\ldots ,s_{D-1}\rangle$$

where *D* represents the number of precursory pattern sequences in the precursory pattern sequence sample in the case where the prediction time range is *N* hours.

For model training, it is necessary to establish the label set corresponding to the precursory pattern sequence set, which can be expressed as:

(4) $$T=\langle t_{0},t_{1},t_{2},\ldots ,t_{D-1}\rangle$$

where $t_{i}$ is the label of the precursory pattern sequence $s_{i}$, and if a large-energy event is about to occur, $t_{i}=1$; otherwise, $t_{i}=0$. Its calculation method is shown as follows:

(5) $$f\left(x\right)=\left\{\begin{array}{c} 0,e_{i}<E\\ 1,e_{i}\geq E \end{array}\right.$$

where $e_{i}$ is the maximum energy value of the precursory pattern sequence $s_{i}$ in the next *N* hours. *E* is the energy threshold for large-energy events, which is generally taken as $5\times 10^{4}$ J or $1\times 10^{5}$ J.

### 3.2. Feature Extraction

#### 3.2.1. Knowledge-Driven Explicit Features

Explicit features are features extracted based on expert knowledge, including the *b* value [28], *a* value [29], A(b) value [30], seismic absence [31], coal burst activity scale ΔF [32], microseismic time information entropy [33], and equivalent energy level parameter EEM [34]. Multiple mine-seismic indicators, such as the seismicity *S* value, are used as explicit feature candidate sets. Considering the influence of factors such as different coal mine geological structures, not all indicators are suitable for coal burst prediction. Therefore, to solve the selection problem of microseismic indicators for different coal mines, this paper designs a feedback selection method of microseismic indicators. The main idea of this method is to dynamically add indicators, and then use the data of other mined-out working faces in the mine to calculate the model accuracy after adding different indicators, and judge whether the indicator set is optimal according to the accuracy. Specific steps are as follows. Assume that the set of microseismic indicators is $\left\{a_{1},a_{2},\ldots ,a_{m}\right\}$; we treat each metric as a subset of candidates and evaluate a subset of m candidate single indicators. Assume that the optimal candidate set in the first round is $\left\{a_{2}\right\}$; we take it as the first round selection set. Next, adding an indicator to this selected set constitutes a candidate subset consisting of two indicators. Suppose that $\left\{a_{2},a_{5}\right\}$ is optimal in the m−1 candidate subsets, and use it as the second round selection set. Until r+1 round, the optimal candidate subset is inferior to the previous round; then, the *r*th round of indicators set is used as the final feature selection result. In this research, the evaluation criterion for evaluating the pros and cons of a feature subset is the average model accuracy of the feature subset on different datasets and different sampling methods. Based on the above method, specific indicators can be screened for different coal mines as display features. The feedback-type microseismic indicator selection mechanism solves the problem of microseismic indicator selection and improves the model prediction accuracy.

#### 3.2.2. Data-Driven Implicit Features

**Figure 3:**
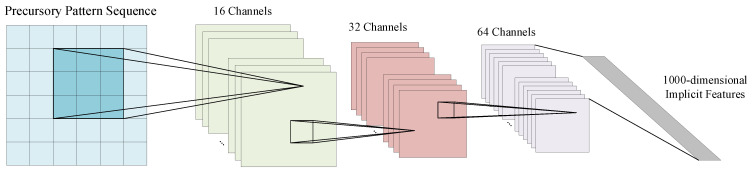
Data-driven feature extraction network based on convolutional neural network.

As shown in Figure 3, in order to mine the hidden laws in the massive microseismic data, we propose an implicit feature extraction method based on deep learning, which uses the convolutional neural network in deep learning to achieve implicit feature extraction. A convolutional neural network is a neural network specially designed to process data with a grid-like structure. Convolutional neural networks have the ability to learn representations [35,36,37,38]. Its artificial neurons can respond to a part of the surrounding units in the coverage area. They are composed of one or more convolutional layers, a fully connected layer at the top, as well as association weights and pooling layers. This structure enables convolutional neural networks to exploit the two-dimensional structure of the input data. The convolution kernel parameter sharing in the hidden layer and the sparsity of the connection between the layers enable the convolutional neural network to achieve implicit feature extraction with a small amount of computation. Therefore, this paper uses a deep convolutional neural network to extract implicit features in massive mine seismic data. The convolutional neural network used is a 3-layer convolution, including 88 convolution kernels, and outputs a 1000-dimensional implicit feature vector that is the implicit feature to be extracted in this paper.

### 3.3. Prediction Module

Based on the training data set, the prediction module generation establishes the objective function, and uses the back-propagation algorithm to update the network weights to minimize the loss of the objective function, thereby generating the prediction module. The trained model can be used for large-energy event prediction, which can predict whether a large-energy event will occur in the future. The prediction module mainly includes two modules: feature fusion and classification network. Section 3.3.1 introduces feature fusion, Section 3.3.2 introduces the classification network, and Section 3.3.3 introduces the model training.

#### 3.3.1. Feature Fusion

In the prediction module, explicit features and implicit features need to be deeply fused. The complexity and heterogeneity of explicit and implicit features make simple weighted feature fusion methods unsuitable. Therefore, this paper uses the attention mechanism method [39] to achieve feature fusion, which can realize the weighting of each dimension within the explicit feature and the implicit feature.

**Figure 4:**
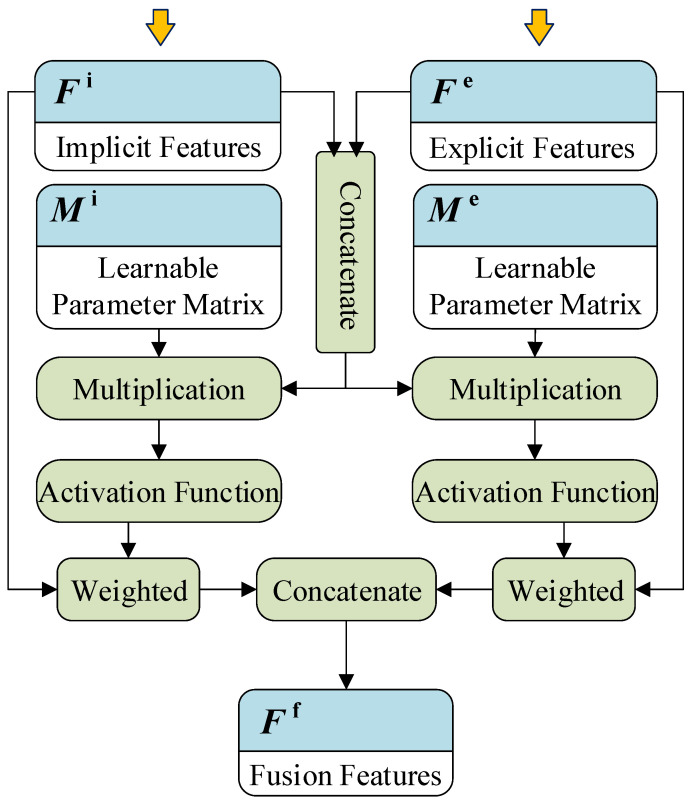
The workflow of the feature fusion process.

As shown in Figure 4, first, the explicit features $F^{e}$ and implicit features $F^{i}$ are merged to obtain the initial feature vector $F^{s}$:

(6) $$F^{s}=\left[F^{e},F^{i}\right]$$

where the dimensions of the explicit and implicit eigenvectors $\left(F^{e}\in R^{d_{1}}\;F^{i}\in R^{d_{i}}\right)$ are $d_{1}$ and $d_{2}$, respectively. The dimension of the fusion feature vector $F^{s}\in R^{\left(d_{1}+d2\right)}$ is $d_{1}+d_{2}$. In the attention mechanism [40,41], the weight vectors of explicit features and implicit features are denoted as $V^{e}\in R^{d_{1}}$, $V^{i}\in R^{d_{2}}$. The calculation method is as follows:

(7) $$\begin{array}{cc} V^{e} & =H(F^{s}\cdot M^{e})\\ V^{i} & =H(F^{s}\cdot M^{i}) \end{array}$$

where $M^{e}\in R^{\left(d_{1}+d_{2}\right)\times d_{2}}$ and $M^{i}\in R^{\left(d_{1}+d_{2}\right)\times d_{2}}$ are two learnable parameter matrices; H(x) is the activation function $\frac{1}{1+e^{-x}}$. Each dimension of the weight vector $V^{e}$ and $V^{i}$ corresponds to the weight of each feature dimension of $F^{e}$ and $F^{i}$. Finally, the final fused feature vector $F^{f}$ is calculated as:

(8) $$\begin{array}{c} F^{f}=\left[F^{e}⊙V^{e},F^{i}⊙V^{i}\right] \end{array}$$

where ⊙ denotes the Hadamard product. The fused features are used as input to the subsequent classification network.

#### 3.3.2. Classification Network

The classification network in the prediction module consists of a fully connected layer and an activation function. In deep learning, the fully connected layer acts as a classifier, which can map the learned distributed feature representation into the sample label space [42,43]. The fully connected layer in this model is composed of 2000 neurons. Then, the activation function is used for normalization to obtain the probability of whether there is a large-energy event. If the probability of a large-energy event is greater than the probability of no large-energy event, the output is 1; otherwise, the output is 0.

#### 3.3.3. Model Training

Considering the low probability of large-energy events, the training data set samples are unbalanced, where the data labeled as small-energy events are much more than those with large-energy events. If the traditional deep learning model training method is used, it will lead to the problem that the model is biased towards learning a class with more samples during classification. To solve this problem, this paper draws on the idea of “re-scaling”. During the training process, the model can dynamically adjust the weight of each class in calculating the loss according to the distribution of batch samples and overall samples. In network training, the weighted cross-entropy loss function is used to calculate the loss value of the model, and by continuously updating the parameters in the neural network model, the loss of the model on the training data set is minimized. In addition, in the prediction task of coal burst, to reduce the false negative rate of events caused by large energy, this paper adds various learning weights, $Z_{0}$ and $Z_{1}$, to the loss function. By adjusting the learning weight of large-energy events, the model can be more biased towards the prediction of large-energy samples, thereby reducing the false negative rate of large-energy events. The weighted cross-entropy loss *L* in this paper can be expressed as:

(9) $$\begin{array}{cc} L & =\frac{1}{N}\sum_{i=1}^{N}L_{i}\\ L_{i} & =\frac{-z_{0}\times w_{0}\times y_{i0}\times ln^{p_{io}}-z_{1}\times w_{1}\times y_{i1}\times ln^{p_{io}}}{w_{0}+w_{1}} \end{array}$$

where $L_{i}$ represents the loss value of the *i*th precursory pattern sequence. *N* is the number of precursory pattern sequences. $z_{0}$ and $z_{1}$ represent the learned weights for the two classes, respectively. $w_{0}$ and $w_{1}$ represent the sample distribution weights of classes 0 and 1, respectively. If the label of the ith precursory mode sequence is a small-energy event, then $y_{i0}=1$, $y_{i1}=0$; otherwise, $y_{i0}=0$, $y_{i1}=1$. $p_{i0}$ is the predicted probability that the observed sample *i* is class 0. $p_{i1}$ is the predicted probability that the observed sample *i* is class 1. The above method effectively improves the problem of unbalanced data categories, effectively accelerates the model convergence speed, and improves the model prediction accuracy.

## 4. Engineering Experiments

In order to verify the effectiveness of the fusion-driven model, a large number of experiments are described in this section using the microseismic data of multiple working faces of the Gaojiapu Coal Mine. First, the overall performance of the model and the influence of different parameters were evaluated by using 13,058 seismic data from May 2019 to May 2020 on the 204 working face of Gaojiapu Coal Mine, and a comparative experiment with the single-driven model was established. In addition, the prediction module was trained using the seismic data of the 204 working face of Gaojiapu Coal Mine, and then the model was used to predict large-energy events on the 301 working face, where coal was being mined. The experimental results show that the prediction module proposed in this paper is still effective in the cross-working face situation.

### 4.1. Evaluation Indicators

In this experiment, the confusion matrix is used to record the model prediction results. If the actual situation is true and the prediction is true, it is recorded as TP (true positive). If the actual situation is true and the prediction is false, it is recorded as FN (false negative). If the actual situation is false and the prediction is false, then we record TN (true negative). If the actual situation is false and the prediction is true, it is recorded as FP (false positive). In the calculation process of the confusion matrix, we first define the “true and false” in the preceding paragraph. Among them, “true” can be expressed as the occurrence of a large-energy event coal burst; “false” means that a large-energy event has not occurred. Here, the confusion matrix of large-energy events is defined.

As shown in Table 1, in the model evaluation, this paper uses three evaluation indicators: accuracy rate (ACC), true case rate (TPR), and false discovery rate (FDR). The accuracy rate is the ratio of the number of events predicted accurately to the total number of events, reflecting the overall performance of the model. The true case rate is the ratio of the number of events that are predicted to be true and are actually true to the number of events that are actually true. This experiment represents the proportion of precursory pattern sequences predicted to be large-energy events in the samples of real large-energy events. The false discovery rate is the ratio of the number of events predicted to be true that are actually false to the total number of events predicted to be true, reflecting the false positive rate of the model. The calculation formula of the above indicators is as follows:

(10) $$\begin{array}{cc} ACC & =\frac{TP+TN}{TP+FP+TN+FN}\\ TPR & =\frac{TP}{TP+FN}\\ FDR & =\frac{FP}{FP+TP} \end{array}$$

### 4.2. Overall Performance

**Figure 5:**
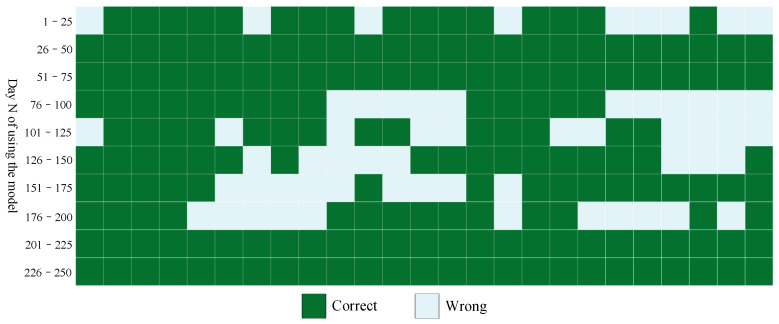
Overall performance.

To evaluate the overall performance of the model, the design ideas of this experiment are as follows: The initial model is trained using data from 1–100 days of working face 204, and then predicts whether large-energy events (energy greater than $1\times 10^{5}$ J) will occur in days 101 to 103. The simulation time goes by 1 day, and the model is updated. We retrain the model with data from days 1–101 and predict whether a large-energy event will occur in days 102–104. The simulation time goes by for another day, and the model is updated again. We retrain the model with 1–102 days of data. Then, we predict whether a large-energy event will occur in days 103 to 105, and so on. Then, a total of 250 simulation predictions were tested. The prediction results are shown in Figure 5; dark green indicates that the model predicts correctly, and light green indicates that the model predicts incorrectly. We can find that as the training data set continues to increase, the prediction accuracy of the model becomes higher and higher. Table 2 shows the overall performance of the model. The accuracy rate reached 76.68%. The true case rate was 73.13%, and the false positive rate was only 19.01%. In conclusion, the fusion prediction module has good performance.

### 4.3. Influence of Different Parameters

This subsection tests the effect of different parameters, including prediction time, sequence length, large-energy event threshold, data sampling time window size, and balance factor, on model performance. The training set and test set are divided using the leave-out method commonly used in machine learning, where the ratio of the training set and test set is 7:3.

#### 4.3.1. Influence of Prediction Time

To evaluate the performance of the model under different prediction time scenarios, the experiments are designed as follows. The sampling time window I=12 h, the precursory pattern sequence length p=28, and the loss function balance factor $z_{0}=0.8$, $z_{1}=1.5$. When the above parameters remain unchanged, we set the prediction time N=24 h, N=48 h, N=72 h, respectively. Two large-energy thresholds are set at each prediction time, $E=5\times 10^{4}$ J and $E=1\times 10^{5}$ J, and we then run the model test. Table 3 shows the experimental results for different prediction times. The results show that this model can effectively predict whether large-energy events will occur in the next 1–3 days. In the case of two large-energy thresholds of $E=5\times 10^{4}$ J and $E=1\times 10^{5}$ J, optimal model performance for N=72 h, and its TPR can reach 81.15%.

#### 4.3.2. Influence of Sequence Length

**Figure 6:**
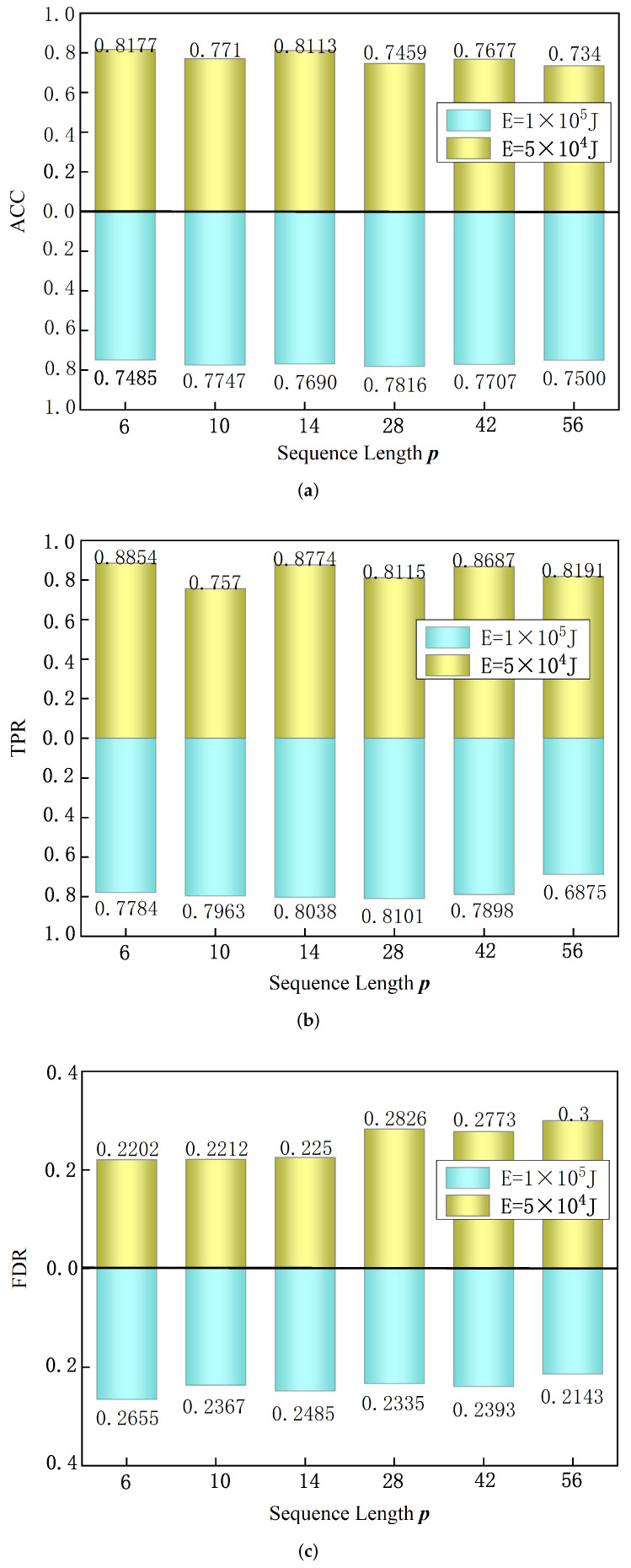
Test results of different sequence lengths including (**a**) ACC, (**b**) TPR, (**c**) FDR.

In order to evaluate the effect of different precursory pattern sequence lengths on model performance, this experiment uses different sequence lengths for model testing. The experimental setup is as follows: sample time window I=12 h, prediction time N=12 h, and loss function balance factor $z_{0}=0.8$, $z_{1}=1.5$. Under the condition that the above parameters remain unchanged, we set the precursory pattern sequence length *p* as 6, 10, 14, 28, 42, 56. We set two large-energy thresholds at each sequence length, $E=5\times 10^{4}$ J and $E=1\times 10^{5}$ J, and then run the model test. The model test results are shown in Figure 6. Experiments show that when a large-energy threshold $E=5\times 10^{4}$ J, a shorter precursory pattern sequence is beneficial to improve the ACC and TPR of the model. The case of p=6 achieved the highest ACC, reaching 81.77%; its TPR reached 88.54%, and the FDR was only 22.02%. When the large-energy threshold $E=1\times 10^{5}$ J, the case of p=28 achieved the highest ACC, reaching 78.16%, and FDR reached 23.35%. To sum up, under the conditions of two large-energy thresholds of $E=5\times 10^{4}$ J and $E=1\times 10^{5}$ J, the optimal sequence lengths *p* are 6 and 28, respectively.

#### 4.3.3. Influence of Time Window Size

**Figure 7:**
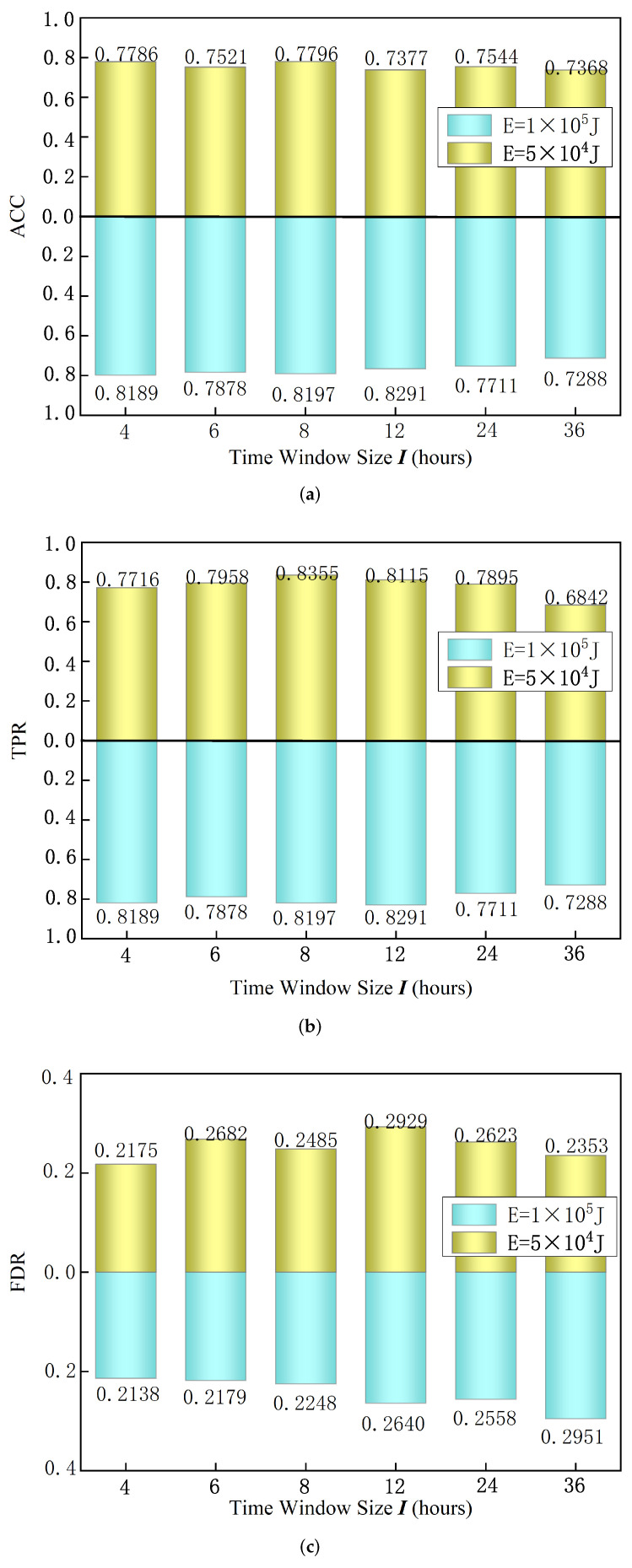
Test results of different time window sizes including (**a**) ACC, (**b**) TPR, (**c**) FDR.

In order to verify the influence of different time window sizes on model performance, this experiment uses different time window sizes for model training. The experimental setup is as follows: precursory pattern sequence length p=28, prediction time N=72 h, and loss function balance factor $z_{0}=0.8$, $z_{1}=1.5$. Under the condition that the above parameters remain unchanged, we set the time window as 4 h, 6 h, 8 h, 12 h, 24 h, 36 h, respectively. Two large-energy thresholds are set under each time window, respectively, $E=5\times 10^{4}$ J and $E=1\times 10^{5}$ J, and we then run the model test. The model test results are shown in Figure 7. The results show that the highest accuracy rate of 77.96% is obtained when the large-energy threshold is $E=5\times 10^{4}$ J, and its time window size I=8 h. The TPR reached 83.55%. When the maximum energy threshold is $E=1\times 10^{5}$ J, the highest accuracy rate is 79.81%; when the time window is I=4 h, the TPR reaches 81.89%, and the FDR is only 21.38%. In summary, under the conditions of two large-energy thresholds of $E=5\times 10^{4}$ J and $E=1\times 10^{5}$ J, the optimal time windows are 8 h and 4 h, respectively.

#### 4.3.4. Influence of Balance Factor

**Figure 8:**
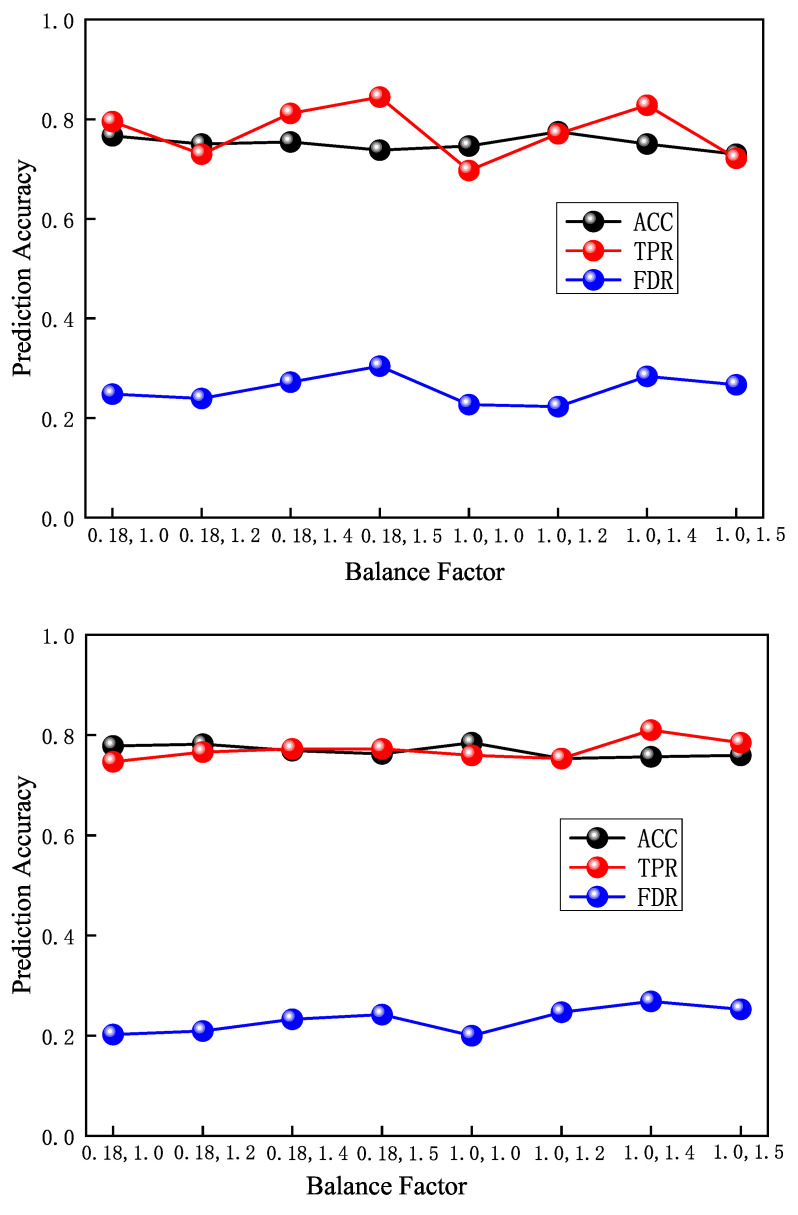
Test results of different balance factors.

In order to verify the influence of the loss function balance factor on the performance of the model, this experiment selects multiple groups of loss function balance factors to train the model. The experimental setup is as follows: time window size I=12 h, precursory pattern sequence length p=28, prediction time N=72 h. With the above parameters unchanged, we set the balance factor $z_{0}=0.8$ and $z_{1}=1.5$ as (0.8,1.0),(0.8,1.2),(0.8,1.4),(0.8,1.5),(1.0,1.0),(1.0,1.2),(1.0,1.4),(1.0,1.5), separately. Two large-energy thresholds are set under each group of balance factors, which are $E=5\times 10^{4}$ J and $E=1\times 10^{5}$ J, and we then perform model testing. The model test results are shown in Figure 8. Experiments show that when the large-energy threshold is $E=5\times 10^{4}$ J and the balance factor is (1.0,1.2), the highest accuracy rate is 77.46%, and its TPR is 77.05%. When the balance factor is (0.8,1.4), the highest TPR is 81.15%, and its accuracy is 75.41%, which is only 1.64% lower than 77.05%. When the large-energy threshold $E=1\times 10^{5}$ J, the model accuracy rates are all between 75% and 79%, and the highest TPR of 81.01% is obtained when the balance factor is (1.0,1.4). To sum up, under the conditions of two large-energy thresholds of $E=5\times 10^{4}$ J and $E=1\times 10^{5}$ J, the optimal selections of the balance factor are (0.8,1.4) and (1.0,1.4), respectively.

### 4.4. Cross-Working Face Performance Test

To evaluate the performance of the prediction model across working faces, this experiment uses data from the Gaojiapu 204 working face to train the model, and then uses the model to test it on the 301 working face that is being mined. The experimental setup is as follows: time window size I=12 h, precursory pattern sequence length p=28, prediction time N=72 h, balance factor (1.0, 1.4). With the above parameters unchanged, $E=5\times 10^{4}$ J and $E=1\times 10^{5}$ J are used as the large-energy thresholds, respectively. The experimental results are shown in Table 4. Experiments show that the accuracy of the model is still above 75% in the case of cross-working face prediction. It can be seen that different working faces in the same mining area have similar characteristics. The prediction model of this paper still has relatively good performance in the case of a cross-working face.

### 4.5. Comparison with the Single-Driven Model

In order to verify the effectiveness of the fusion-driven prediction module proposed in this paper, the data of the 204 working face were selected for model training. The experimental parameters are set as follows: time window size I=12 h, precursory pattern sequence length p=28, prediction time N=72 h, balance factor (1.0, 1.4). Under the conditions of large-energy thresholds $E=5\times 10^{4}$ J and $E=1\times 10^{5}$ J, respectively, we trained three models of “data-driven + knowledge-driven”, “data-driven”, and “knowledge-driven”. As shown in Table 5, experiments show that the fusion-driven model has an accuracy rate of over 75% and a TPR of over 81% under two large-energy thresholds. The accuracy of the “knowledge-driven” models is between 60% and 65%. Although the accuracy of the “data-driven” model has also reached more than 70%, its TPR is approximately 7% lower than that of the “fusion-driven” model. Therefore, the “fusion-driven” model is significantly better than the “knowledge-driven” model, and the prediction effect of large-energy events is improved relative to the “data-driven” model. It can be seen that the “fusion-driven” model has a significant improvement in the prediction accuracy compared with the single-driven model.

## 5. Conclusions

This paper presents FDNet, which is a knowledge- and data fusion-driven deep neural network for coal burst prediction. First, we design an expert knowledge indicator selection method based on a subset search strategy to solve the multi-indicator screening problem of mining microseismic data. Then, we establish a mine microseismic data extraction method based on a deep convolutional neural network, realizing the implicit feature extraction of massive mine microseismic data. Finally, we propose a feature deep fusion method of mine microseismic data based on an attention mechanism, which realizes the feature fusion based on knowledge-driven and data-driven aspects. In addition, we have conducted a set of engineering experiments to evaluate the performance of FDNet. We have evaluated the impact of different factors and compared the model with the state-of-the-art method. The results show that FDNet has good prediction accuracy and robustness. However, FDNet is not applicable to all coal mines, so we will conduct research on the model’s generalization ability in future work.

## Figures and Tables

**Table 1:** Confusion matrix.

		Actual	
		True	False
**Predicted**	True	True–True (*TP*)	True–False (*FP*)
	False	False–True (*FN*)	False–False (*TN*)

**Table 2:** Overall performance test results.

Evaluation Metrics	Result
ACC	0.7668
TPR	0.7313
FDR	0.1901

**Table 3:** Influence of prediction time.

Prediction Duration N(h)	Large Energy Threshold (J)	ACC	TPR	FDR
24	5 × 104	0.6993	0.8601	0.3492
	1 × 105	0.7386	0.6932	0.2375
48	5 × 104	0.7358	0.813	0.2958
	1 × 105	0.7519	0.7481	0.2462
72	5 × 104	0.7459	0.8115	0.2826
	1 × 105	0.7658	0.8101	0.2558

**Table 4:** Cross- working face performance test results.

Large Energy Thresholds (J)	ACC	TPR	FDR
5 × 104	0.7664	0.75	0.2245
1 × 105	0.7521	0.6581	0.1895

**Table 5:** Test results of single-driven model and fusion-driven model.

Model	Fusion-Driven		Data-Driven		Knowledge-Driven	
Large-energy threshold (J)	5 × 10^4^	1 × 10^5^	5 × 10^4^	1 × 10^5^	5 × 10^4^	1 × 10^5^
ACC	0.7500	0.7563	0.7400	0.7532	0.6475	0.6234
TPR	0.8279	0.8101	0.7623	0.7405	0.6721	0.7722
FDR	0.2837	0.2686	0.256	0.2403	0.3594	0.4049

## Data Availability

Not applicable.
